# Albumin and the fibrinogen-to-albumin ratio: Biomarkers for the acute phase response following total knee arthroplasty

**DOI:** 10.1371/journal.pone.0247070

**Published:** 2021-02-16

**Authors:** Emilie Amaro, Stephanie N. Moore-Lotridge, Bronson Wessinger, Michael A. Benvenuti, Thomas J. An, William K. Oelsner, Gregory G. Polkowski, Jonathan G. Schoenecker

**Affiliations:** 1 Department of Orthopedics, Vanderbilt University Medical Center, Nashville, Tennessee, United States of America; 2 Vanderbilt Center for Bone Biology, Vanderbilt University Medical Center, Nashville, Tennessee, United States of America; 3 School of Medicine, Vanderbilt University Medical Center, Nashville, Tennessee, United States of America; 4 Department of Pathology, Microbiology, and Immunology, Vanderbilt University Medical Center, Nashville, Tennessee, United States of America; 5 Department of Pharmacology, Vanderbilt University Medical Center, Nashville, Tennessee, United States of America; 6 Department of Pediatrics, Vanderbilt University Medical Center, Nashville, Tennessee, United States of America; Cleveland Clinic, UNITED STATES

## Abstract

**Purpose:**

Complications following total knee arthroplasty (TKA) lead to patient morbidity and cost. While acute phase reactants, such as c-reactive protein (CRP) and fibrinogen, have been used to predict complications following TKA, the extent and duration of changes in albumin levels following TKA are unknown. It is hypothesized that like CRP and fibrinogen, albumin, and the fibrinogen/albumin ratio (FAR) represent useful measures of the acute phase response (APR) following TKA. The purpose of this study was to describe the longitudinal course of albumin and FAR in healthy patients following TKA, relative to established biomarkers, and examine if the variance in albumin or FAR correlates with patient comorbidities.

**Methods:**

This retrospective cohort study of patients undergoing TKA at a tertiary medical center. CRP, fibrinogen, and albumin values were collected pre- and post-operatively. An age-adjusted Charlson comorbidity index (CCI) was utilized as a measure of patient comorbidity status.

**Results:**

The median preoperative albumin value was 4.3 g/dL, which dropped to 3.6 g/dL on postoperative day 1 following TKA. The albumin value returned to 93% of the baseline by postoperative week 2. The course of albumin inversely mirrored the course of CRP (r = -0.41). Median preoperative FAR was 0.087 g/L, which rose to 0.130 g/L by postoperative week 2 and returned to baseline by postoperative week 6. While preoperative FAR strongly correlated with postoperative week 2 values (r = 0.74), there was a weak positive correlation between age-adjusted CCI and pre-operative FAR (r = 0.24) in patients undergoing primary TKA.

**Conclusion:**

Albumin levels follow a predictable postoperative decline that inversely correlates with CRP in healthy patients following TKA. Given the low cost and abundance of laboratories offering albumin levels, direct albumin levels and/or albumin ratios such as FAR may be underutilized biomarkers for monitoring the APR following TKA.

## Introduction

Complications following total knee arthroplasty (TKA) lead to high patient morbidity and markedly increased healthcare costs. Periprosthetic joint infections, for example, typically require multiple surgical revisions, prolonged antibiotic therapy, and occasionally amputation [1]. The treatment of an infected total knee implant costs 3 to 4 times that of an uncomplicated primary TKA [2]. Much work has been invested in discovering prognostic biomarkers that predict postoperative complications early in their course.

The acute phase response (APR) is the body’s response to tissue injury. Cytokines are released at the site of tissue injury and travel to the liver and other organs where thousands of genes are regulated to produce proteins that aid in tissue survival and regeneration [3, 4]. Therefore, acute phase reactants in circulation are sensitive, albeit nonspecific, laboratory measures indicating tissue injury. Studies have shown that C-reactive protein (CRP) and fibrinogen levels rise rapidly with the onset of tissue damage, with CRP falling rapidly following injury resolution and fibrinogen showing a more prolonged descent to baseline value [5–7]. CRP levels remain elevated in the face of repeated tissue injury and therefore can be utilized as a marker of injury duration [8]. Prior studies have demonstrated the clinical utility of serially assessing CRP following surgeries, such as total joint arthroplasties, to predict patient outcomes and assess the risk of complications [9, 10].

While most plasma acute phase reactants increase in response to injury, the concentration of several reactants decreases following tissue injury. These proteins are referred to as negative acute phase reactants. Albumin is a known negative acute phase reactant that decreases in response to physiologic stress. Clinically, albumin levels are often used as a serologic marker of nutritional status, with low albumin levels representing malnutrition, although several studies have shown that albumin is an inconsistent measure of nutritional status [11–14]. Furthermore, decreased albumin levels are predictive of morbidity and mortality postoperatively, as well as in disease states such as cirrhosis, renal failure, burns, malignancy, and autoimmune disorders [15–23]. Similarly, Huang *et al* and others have demonstrated that preoperative hypoalbuminemia, defined as an albumin level less than 3.5 g/dL, correlates with postoperative complications in patients undergoing a TKA [24–27]. While these studies illustrates the potential clinical values for measuring albumin preoperative, the value of longitudinal measure of albumin following TKA to predict patient outcomes from surgery or complications remains unknown.

While individual serologic measures of the APR can prognosticate disease severity and patient outcomes, recent studies have begun to demonstrate the improved predictive capacity of combinatory measures. For example, the fibrinogen to albumin ratio (FAR) has been demonstrated to significantly correlate with poorer survival and negative outcomes in patients suffering from cancer or cardiovascular disease [28–33]. To date, limited investigations in the orthopaedic space have reported on the clinical utility of serial measures of either albumin or FAR. Therefore, the purpose of this study was to describe the normal course of albumin and FAR in healthy patients following TKA, relative to the course of known plasma acute phase reactants, CRP, and fibrinogen. Secondarily, this study will examine if the variance in preoperative albumin or FAR correlates with patient comorbidities, as measured by the Charlson comorbidity index (CCI). We postulate that albumin levels and FAR will follow a defined and predictable course following acute surgical intervention. Secondarily, we hypothesize that CCI will positively correlate with preoperative FAR.

## Material and methods

### Study design

Following approval by the Vanderbilt University Institutional Review Board (IRB), this retrospective cohort study was conducted on patients undergoing primary TKA at a tertiary medical center from January 2014 to May 2016. Given the retrospective nature of this study, written or oral consent from the patient was waved. All surgeries included in the study were conducted by a single surgeon (GGP). CRP, fibrinogen, and albumin values during hospitalization and in the postoperative period were obtained from the electronic medical record.

### Inclusion and exclusion criteria

The study included adult patients (>18 years of age) undergoing primary, unilateral TKA for osteoarthritis from January 2014 to May 2016. The age-adjusted Charlson Comorbidity Index, which accounts for 19 preoperative comorbidities, was calculated for each patient in the study. Patients were excluded from the study if they underwent atypical or complex primary arthroplasty. This included procedures for post-infectious arthritis, post-traumatic osteoarthritis, osteonecrosis, and rheumatoid arthritis. Furthermore, patients with postoperative complications such as joint loosening, infection, and wound dehiscence were also excluded from the study, as the purpose of this study was to determine the laboratory change of albumin following an uncomplicated total knee arthroplasty.

A subgroup comparison of excluded patients with rheumatoid arthritis or a prosthetic joint infection (conducted by the same surgeons within the study period) were examined in comparison to included patients.

### Laboratory values

CRP, fibrinogen, and albumin laboratory values were collected preoperatively, during the hospitalization, and postoperatively. The preoperative period was defined as within 60 days before surgery; postoperative day 0 (POD0) was defined as the same calendar date, yet following the start time of the procedure; postoperative day 1 (POD1) was defined as the calendar date following the procedure; postoperative day 2 (POD2) was defined as two calendar dates following the procedure; postoperative day 3 (POD3) was defined as three calendar dates following the procedure; week 2 was defined as postoperative day 10 to 20; and week 6 was defined as postoperative day 35 to day 50. These ranges were selected due to variability in clinic follow-up. Only two patients had a POD2 albumin value measure, and none had a POD3 albumin value measure. Therefore, these time points were not included in the albumin or FAR subgroup analysis, although they were included for the CRP and fibrinogen subgroups. FARs were calculated and presented in g/L.

Raw data for included and excluded patients can be found in the S1 Data.

### Statistical analysis

A correlation coefficient test was performed in MATLAB (Natick, MA) to determine the association between the postoperative laboratory values. GraphPad V9.0 (College Station, TX) was utilized to visualize data throughout and conduct non-parametric t-test assessments between excluded and included patient cohorts.

## Results

One hundred ninety-nine total patients underwent primary TKA during the time period of the study. One hundred sixty-five patients were included in the study, including 62 males and 103 females. The average age of all patients was 64.7 years (range: 35.5–89.7 years). The average age-adjusted Charlson Comorbidity Index of all patients was 2.19 (Table 1). The average estimated blood loss during the surgery within this cohort was 142.7 mL, with an average change in hemoglobin from the preoperative period to POD0 of 13.1% or 17.2% by POD1.

**Table 1:** Demographics of patients included in the study.

Characteristic	TKA (n = 165)
Age at time of surgery (y), mean (range)	64.7 (35.5–89.7)
Male, n (%)	62 (37.6)
Age-adjusted CCI, mean (SD)	2.19 (2.13)

All patients underwent a primary TKA and had an uncomplicated postoperative course. SD, standard deviation.

### CRP

**Fig 1:**
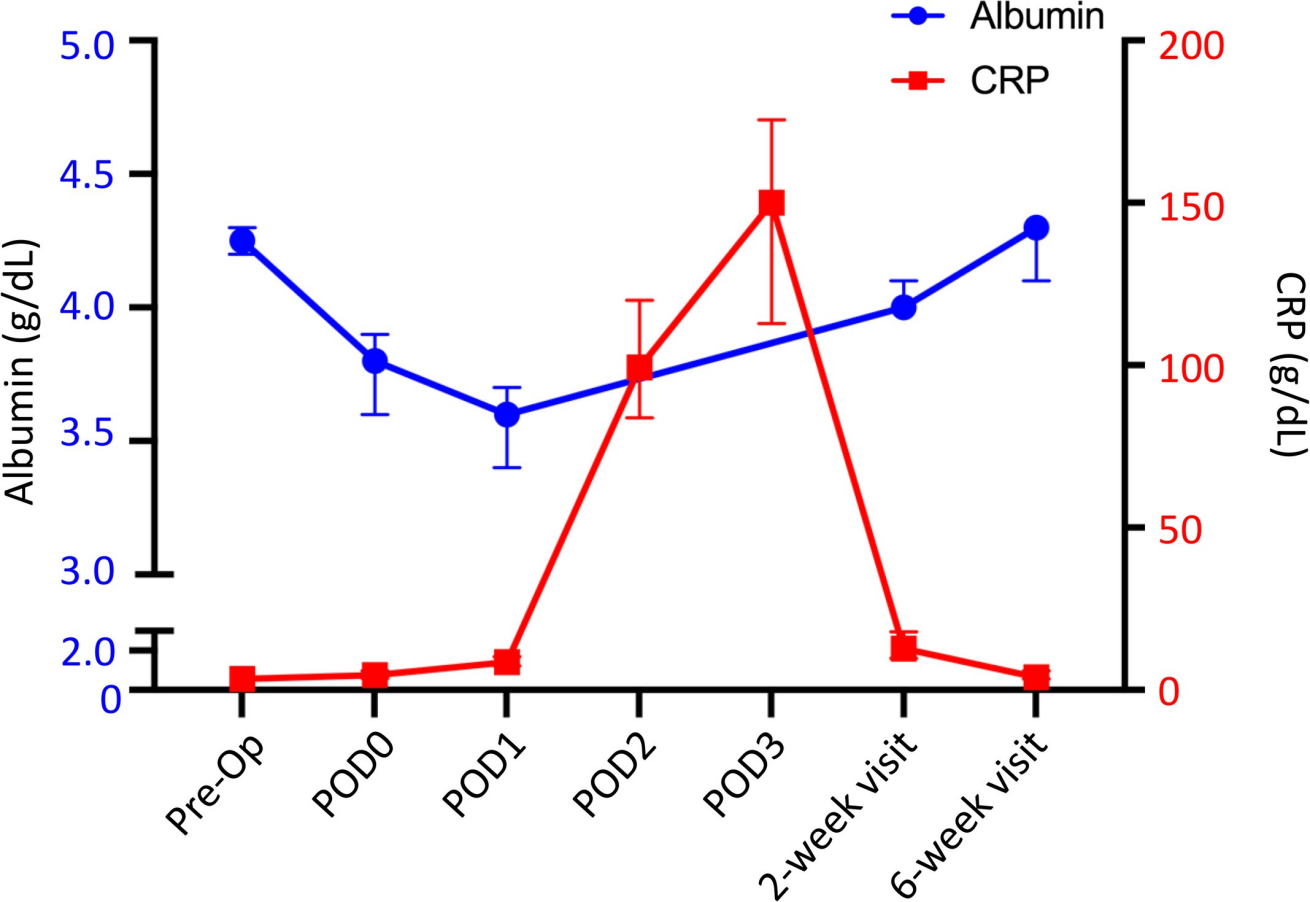
Median circulating albumin (blue circle) and CRP (red square) following TKA. Y-error bars depict a CI of 95%. r = -0.41.

The median preoperative CRP value was 3.4 mg/L, which increased to 153.0 mg/L on POD3. The CRP value nearly returned to baseline (median 12.8 mg/L) by postoperative week 2 and was entirely within normal limits by postoperative week 6 (median 4.0 mg/L) (Fig 1, Table 2).

**Table 2:** Circulating acute phase reactants following TKA.

	Pre-Op	POD0	POD1	POD2	POD3	2-week	6-week
Albumin (g/dL)	4.3 (4.1–4.5), 150n	3.8 (3.6–3.9), 29n	3.6 (3.4–3.7), 29n	-	-	4.0 (3.9–4.2), 46n	4.3 (4.0–4.4), 55n
CRP (mg/L)	3.4 (1.5–6.9), 157n	4.7 (1.9–9.3), 126n	8.6 (4.8–16.0), 157n	99.4 (60.4–144.0), 132n	153.0 (96.8–187.7), 52n	12.8 (6.7–29.3),	4.0 (2.5–8.5), 99n
Fibrinogen (mg/dL)	386 (333–443), 155n	345 (305–396), 126n	340 (299–392), 157n	480 (424–548), 127n	579 (516–654), 52n	522 (457–601), 125n	391 (355–452), 97n
FAR (g/L)	0.087 (0.076–0.105), 139n	0.099 (0.083–0.113), 29n	0.099 (0.087–0.116), 28n	-	-	0.130 (0.116–0.153), 34n	0.098 (0.086–0.111), 29n

Values presented as median (25^th^-75^th^ interquartile range).

### Albumin

The postoperative change in albumin occurred within the same timeframe as CRP. The median preoperative albumin value was 4.3 g/dL, which dropped to 3.8 g/dL on POD0 (9.2% drop) and 3.6 g/dL (17.5% drop) on POD1. The value returned to 93% of the baseline value by postoperative week 2 (median 4.0 g/dL) and returned to baseline by postoperative week 6 (median 4.3 g/dL) (Fig 1, Table 2).

### Fibrinogen

**Fig 2:**
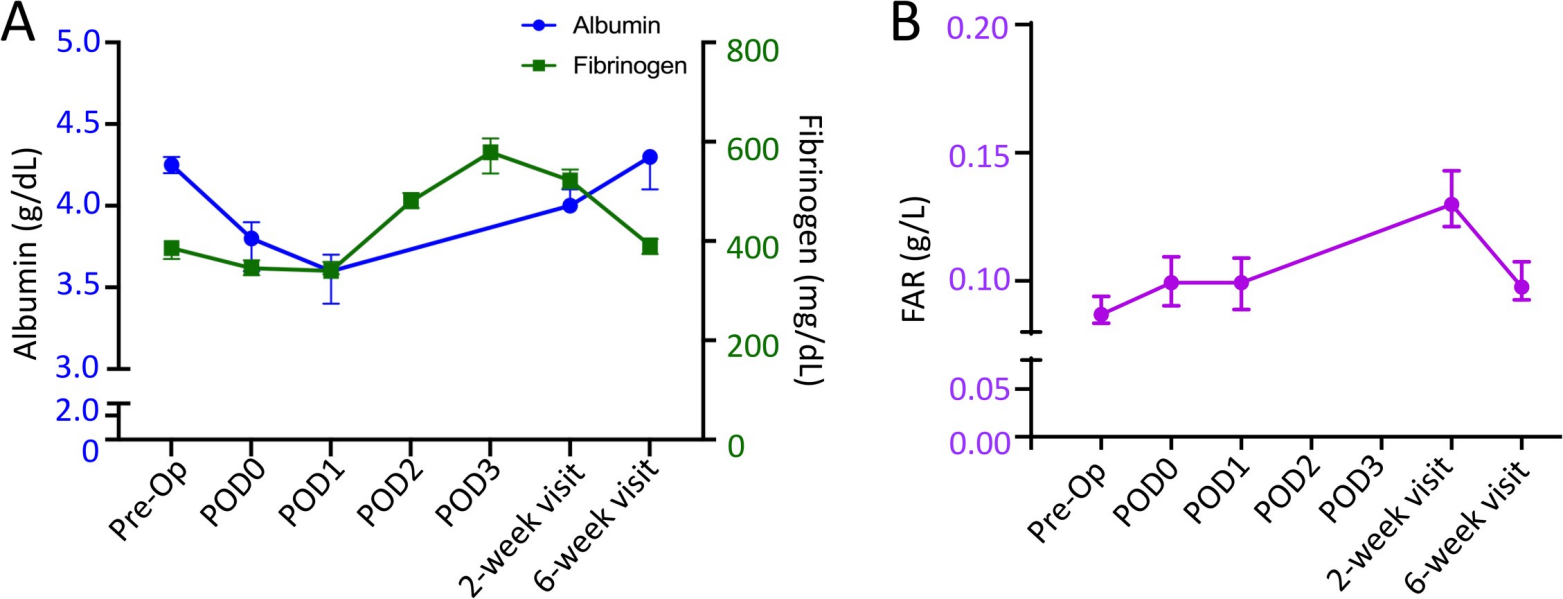
A) Median circulating albumin (blue circle) and fibrinogen (green square) following TKA. r = 0.29. B) Median fibrinogen/albumin ratio (FAR) following TKA. Y-error bars depict a CI of 95%.

Fibrinogen is a long-acting plasma acute phase reactant, with a longer elevation in levels following iatrogenic injury. The median preoperative fibrinogen value was 386 mg/dL, which increased to 579 mg/dL on POD3. The fibrinogen value remained elevated (median 522 mg/dL) at postoperative week 2 but returned to baseline by postoperative week 6 (median 391 mg/dL) (Fig 2A, Table 2).

### Fibrinogen/albumin ratio (FAR)

In adults undergoing uncomplicated primary TKA, the median preoperative FAR was 0.087 g/L. FAR remain consistent though POD1, but was elevated to 0.130 g/L (149% increase from pre-operative values) by postoperative week 2. Aligning with trends in fibrinogen and albumin, FAR levels returned to baseline by postoperative week 6 (median- 0.098 g/L) (Fig 2B, Table 2)

### Correlation

The correlation coefficient between the postoperative course of albumin and CRP was -0.41, while the correlation coefficient between the postoperative course of albumin and fibrinogen was -0.28. There was a strong positive correlation between preoperative FAR and postoperative week 2 values (r = 0.74). Finally, while no correlation existed between preoperative CRP and age-adjusted CCI (r = 0.03), there was a weak positive correlation between age-adjusted CCI and pre-operative FAR (r = 0.24) in patients undergoing primary TKA.

### Case examples of variance

**Fig 3:**
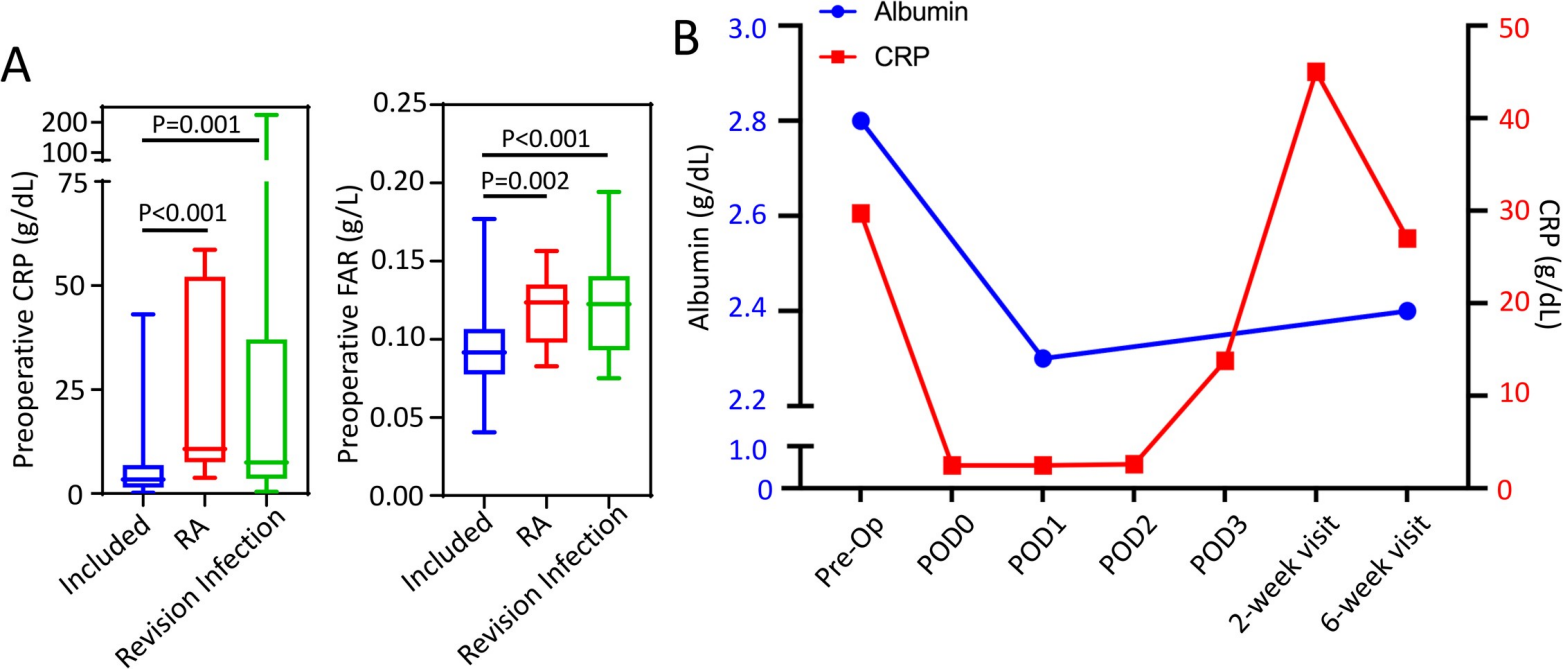
A) Comparison of preoperative CRP and fibrinogen/albumin ratio (FAR) in included study patients (N = 157) and those excluded for rheumatoid arthritis (N = 9) or necessitating a revision TKA due to periprostatic infection (n = 32). Whiskers represent the range of data. Cohorts were compared using a non-parametric ANOVA (Kruskal Wallis test) given that data was nonnormally distributed, α = 0.05. B) Median circulating albumin (blue) and CRP (red) following TKA in a patient with cirrhosis who developed wound dehiscence at postoperative week 2. Y-error bars depict a CI of 95%.

To identify the normal albumin and FAR course following primary TKA, patients with chronic conditions or those undergoing TKA revisions were excluded from this study. In patients excluded, we observed significantly greater preoperative CRP and FAR in patients with either a chronic inflammatory condition (rheumatoid arthritis) or those necessitating a revision TKA for a periprosthetic infection (Fig 3A). Yet, no significant difference in age-adjusted CCI was observed between included patients and excluded patients (p>0.05).

A further example of the variation of the APR following primary TKA was observed in a patient excluded from our study due to the occurrence of a postoperative complication. This patient had advanced cirrhosis with a preoperative albumin level of 2.8 g/dL, a preoperative CRP value of 29.7 mg/L, and a FAR of 0.124 g/L, indicating end-stage disease with a low hepatic synthetic capacity. On POD1, the albumin level decreased 17.8%, to a value of 2.2 g/dL, resulting in a reduced FAR of 0.084 g/L; while the CRP value decreased to 2.5 mg/L (Fig 3B). The CRP value increased dramatically to 45 mg/L at 2 weeks postoperative when the patient presented with wound dehiscence and persistent serous drainage from the surgical incision that ultimately required surgical revision. The patient’s albumin and FAR level remained depressed throughout the postoperative course.

## Discussion

Through serial measures, our data demonstrated that albumin levels decline acutely following TKA, with levels reaching a trough on POD1 and recovering by 6 weeks postoperative. We also have shown that CRP and fibrinogen values increase following TKA and reach a peak on POD3. CRP levels return to baseline by 2 weeks postoperative while fibrinogen levels remain elevated until 6 weeks postoperative. Aligning with the changes in fibrinogen, FAR remains consistent through POD1, but is markedly increased by 2 weeks postoperative with resolution by 6 weeks postoperative. Thus, the course of CRP closely mirrors that of albumin, although CRP production is upregulated while albumin production is downregulated (Fig 1, Table 2). Finally, assessment of excluded patients suggests that elevated preoperative FAR or a persistent depression of albumin outside of the expected time course may be associated with patient comorbidities or complications.

The progression of CRP has been well characterized following total joint arthroplasty and has been shown to follow a predictable course [6]. Persistent elevation in CRP outside of the normal time course is associated with complications, such as periprosthetic infections [34]. Defining the normal course of CRP following arthroplasty has allowed physicians to routinely trend values postoperatively to assess for persistent elevations suggestive of postoperative complications. We have demonstrated that albumin levels similarly have a predictable postoperative course following TKA across 165 patients. Therefore, future studies are warranted to determine whether derangements of albumin or FAR from this natural course are predictive of postoperative complications.

The physiologic role of albumin is multifaceted and includes binding and transporting fatty acids, ions, and amino acids along with maintaining colloid osmotic pressure [15, 18, 35]. Albumin has also been shown to scavenge free radicals and to inhibit platelet function and thrombosis [15]. Synthesis is primarily regulated by colloid osmotic pressure and the osmolality of the extravascular space although growth factors such as insulin, cortisol, and thyroxine stimulate its synthesis [15, 36]. Albumin catabolism occurs primarily in the vascular endothelium and the metabolic half-life of albumin is 17–19 days [15, 18]. Changes in albumin levels have long been attributed to changes in nutritional status. In rats and rabbits, fasting for both short and prolonged periods of time are associated with decreased albumin synthesis within hepatocytes [37–39]. Albumin synthesis declines by 53% in well-perfused hepatocytes in rabbits following an 18–36 hour fast [37]. However, several studies have determined that serum albumin is not a consistent marker of nutritional status in humans. Smale *et al* demonstrated that serum albumin levels in humans remain constant following a 7-day fast [40]. Similarly, a recent study has also shown that albumin levels do not correlate with lean mass, appendicular skeletal mass, or body mass index in the elderly population [11].

The relationship between albumin and malnutrition becomes further complicated in patients with end-stage organ disease or chronic inflammatory conditions. In patients with end stage renal disease, both malnutrition and inflammation lead to hypoalbuminemia [12, 41]. These processes are intricately linked, as several acute phase reactants both suppress appetite and increase protein catabolism [16]. Studies have shown that CRP values and the normalized protein catabolic rate, which represents nutritional status, inversely correlate with serum albumin concentrations [12]. Furthermore, IL-6 has been shown to directly reduce the expression of albumin mRNA in hepatocytes [15]. Thus, in patients with end-stage organ disease, hypoalbuminemia may represent a chronic inflammatory state compounded by malnutrition.

Several studies have shown that decreased albumin is not only predictive of mortality and complications in critically ill patients but also that preoperative and postoperative albumin levels correlate with postoperative complications. One study demonstrated that albumin levels decline as early as 6 hours postoperatively following intraabdominal procedures and that the decrease in albumin correlated with both length of hospital stay and postoperative complications [21]. Ryan *et al* demonstrated that a postoperative albumin level lower than 2 g/L correlated with a two-fold risk of a significant postoperative complication following esophagectomy [23]. Our data demonstrates that, compared to preoperative values, albumin levels drop 17.5% on the first postoperative day following a TKA. This temporal relationship is critical, as healthy patients undergoing significant procedures, such as a TKA, can experience a decline in albumin concentration following injury. This suggests that depression in albumin levels following an injury are not specifically associated with a change in nutritional status; rather, they are a normal response to tissue injury and repair.

In addition to the numerous studies assessing changes in single acute phase reactants relative to disease progression, recent studies in critically ill patients, cardiovascular medicine, and oncology have likewise begun to examine the prognostic value of ratioed measures, such as CRP/albumin and fibrinogen/albumin. Park *et*. *al*. demonstrated that CRP/albumin ratios performed better than CRP alone for predicting mortality in critically ill ICU patients [42]. Likewise, Hwang *et*. *al*. and others have illustrated that FAR has improved prognostic capacity over that of a single marker for prognosis [28] and overall survival of cancer patients [29–31]. To date, few studies have examined ratioed measures of acute phase reactants, such as FAR, in orthopaedic patients. A recent study by Liu *et*. *al*. is one of the first to examine FAR in pathology affecting the musculoskeletal system, where FAR was found to increase in patients with ankylosing spondylitis relative to disease severity [43]. This present study aimed at defining the normal albumin and FAR course following TKA in healthy patients. Aligning with changes in fibrinogen, FAR peaked by 2 weeks and resolved by 6 weeks postoperatively. In patients excluded due to comorbidities or complications, we observed a significant elevation in the preoperative FAR as well as deviation from the normal course. Future studies are warranted to determine whether derangements of FAR from expected preoperative values or the postoperative course are predictive of postoperative complications following orthopaedic surgeries.

The Charlson comorbidity index (CCI) was developed to objectively estimate a patient’s 1-year mortality rates based on preexisting comorbidities [44, 45]. From this initial study, the CCI has been broadly applied across medicinal specialties to predict patient outcomes following interventions. While studies have found that CCI positively predicts patient outcomes following the medical intervention [46–50], studies have likewise reported that CCI had poor or no predictive capacity for patient outcomes or disease severity [51–55]. This present study found that a very weak positive correlation between age-adjusted CCI and pre-operative FAR, yet no correlation between age-adjusted CCI and preoperative CRP. Furthermore, there were no significant differences in age-adjusted CCI between patients included and those excluded for rheumatoid arthritis or revision surgery due to infection; although the risk for complications was higher amongst the excluded patients [56–58]. Together, this data aligns with recent reports suggesting that CCI may not be a sensitive predictor to patient postoperative outcomes following primary total joint arthroplasty [53]. Given the preoperative variance in CRP and FAR between included and excluded patient cohorts, future prospective studies are warranted to examine the positive predictive capacity of preoperative CRP and FAR to predict adverse postoperative outcomes.

This study had several limitations that influence the interpretation of the results. Changes in albumin following surgical procedures may be due to several factors including inflammation, hypermetabolism, redistribution of albumin to the extravascular compartment, changes in colloid osmotic pressure, or blood loss with subsequent hemodilution [15, 18]. The largest confounder in our study is intraoperative blood loss with subsequent fluid repletion and hemodilution. The average change in hemoglobin levels within the albumin cohort from the preoperative period to POD1 closely mirrored the average change in albumin, indicating that hemodilution may play a significant role in the decline in albumin levels we observed. However, several studies have demonstrated similar declines in albumin concentration without significant blood loss and hemodilution. Analysis of decreased albumin values following uncomplicated aortic surgery demonstrated that 18% of the decline in albumin was attributable to blood loss, 6% was attributable to catabolism, and 77% was attributable to redistribution in the extravascular space [59]. Furthermore, regardless of the cause of the convalescent change in albumin, it is clear that the postoperative change in albumin closely mirrors that of CRP, highlighting its role in the acute phase response. Another limitation of this study is that there were limited data points to describe albumin, and thus FAR, on POD2 and POD3. Further large-scale studies are warranted to further characterize the full time course of albumin and FAR in the postoperative setting and the overall clinical utility of these measures to predict patient outcomes.

## Conclusions

This study demonstrates that albumin levels decline rapidly in healthy patients following TKA. Although preoperative albumin levels are predictive of postoperative complications [24], postoperative hypoalbuminemia occurs normally following TKA and follows a predictable postoperative course that mirrors the course of CRP. Elevations in preoperative CRP and FAR are associated with patient statuses more prone to postoperative complications. Therefore, further studies are warranted to determine if persistent depressions of albumin levels or elevated FAR outside of the predictable postoperative course are likewise indicative of complications. Given the low cost and availability of albumin measurement, albumin, and albumin-based ratios such as FAR may be useful serologic measures for monitoring patients’ plasma APR following surgery.

## Supporting information

S1 Data Deidentified raw study data can be found in S1 Data. (XLSX)
